# Time Well Spent? Time Use for Health Information Management Across Home Care Roles: A Cross-Sectional Survey Study in Finland

**DOI:** 10.5334/ijic.9881

**Published:** 2026-09-08

**Authors:** Inka Sylgren, Esa Jämsen, Paulus Torkki

**Affiliations:** 1 University of Helsinki, Helsinki, Finland; 2 Helsinki University Hospital, Helsinki, Finland; 3 LUT University, Lappeenranta, Finland

**Keywords:** integrated care, care coordination, information management, time use, older adults, multimorbidity, home care

## Abstract

**Introduction:**

The growing number of older adults with multiple long-term conditions increases the need for better vertical integration of providers, especially in home care. Ensuring information continuity and care coordination is essential but remains time-consuming for home care professionals (HCPs).

**Methodology:**

This study aimed to quantify HCPs’ time on health information management (HIM) and examine factors associated with this time use. We conducted an exploratory cross-sectional survey in four Finnish home care organizations. Respondents estimated weekly time use for five predefined tasks related to information retrieval, care coordination, and communication. Descriptive statistics, ANOVA, and multiple linear regression examined total HIM time variation.

**Results:**

Based on 625 responses, HCPs averaged 258 minutes weekly on HIM tasks. On average, highest time use was reported by registered nurses (518 minutes) and physicians (336 minutes). Across measured tasks, the largest share of the total time was spent on information retrieval (32.0–46.5%). Profession and direct patient care were the only factors associated with time use, regardless of the region’s integration maturity.

**Conclusion:**

HCPs spend substantial time managing care information. Our findings highlight the critical need for better-integrated systems to enhance care coordination, reduce administrative burden, and enable professionals to devote more time to direct care.

## Introduction

The prevalence of multimorbidity, the coexistence of multiple chronic conditions, is rising worldwide as populations age [1,2]. Caring for multimorbid individuals is inherently complex, and the involvement of multiple providers further increases this complexity, frequently leading to fragmented care [3,4,5]. Care fragmentation compromises quality of care, increases risks of medical errors, and reduces efficiency in already resource-strained systems [6,7,8]. Integrated care has been promoted internationally as a strategy to address these risks by strengthening coordination and continuity, particularly for older adults with complex needs such as multimorbidity [9].

Integration can be examined across horizontal (collaboration at the same level of care) and vertical dimensions (linkages across levels of care) [10]. Both are supported by functional integration (e.g., interoperable information systems, aligned workflows) and normative integration (e.g., shared values, common goals) [11]. When vertical integration is weak, multimorbid individuals remain vulnerable to disrupted information flow and unclear responsibilities [3,12]. These challenges are universal, and countries across Europe and beyond report persistent barriers to connecting home and community-based services with higher-level care [13].

Finland provides a relevant case study. While horizontal integration of health and social services supports older adults’ ability to live at home, vertical integration with other providers such as emergency care, hospitals, primary care, and social services can remain challenging [12,18]. Home care professionals (HCPs) such as registered nurses and practical nurses engage in continuous documentation as part of their daily work, including visit notes, medication management, functional assessments, and care planning [14]. Care planning encompasses the assessment and arrangement of required services and guides the work of the entire care team. Limited vertical integration complicates these tasks: HCPs often lack timely access to updated information such as hospital discharge summaries, medication changes, or other care updates. They frequently encounter fragmented records, incomplete documentation, and misaligned workflows that make caregiving and planning challenging [12,16,17]. Depending on the organization of the home care service, similar challenges may appear in horizontal integration, especially when physician or social work services are organized outside the core team, which typically consists of practical nurses and registered nurses. This creates invisible work in retrieving, verifying, and coordinating information, which can consume substantial time but is rarely measured or acknowledged [18]. Much of this work is mediated through electronic health record (EHR) systems, which are intended to ease the workload but can add to it when functioning poorly [17].

Time use offers a practical indicator of integration gaps and information system inefficiencies [19]. While hospital-based studies show that physicians spend large portions of their working time on EHRs [20], evidence from home care remains limited. Previous studies have examined time use in broad task categories [14,21,22,23,24] but not specifically for information continuity or care coordination. In Finnish home care, for example, registered nurses allocate far more time to documentation than practical nurses (25.2% vs. 3.7%), suggesting that information management burdens may vary meaningfully across roles [24]. Yet how much of this reflects information management across care levels remains unknown. No studies have quantified time spent on information continuity across HCP roles in home care, and little is known about whether demographic characteristics or regional EHR variation contribute to differences in HIM time use, even though skills and experience, including the ability to gather and utilize health data and navigate EHR systems, can be expected to influence the time HIM requires.

Multimorbid clients in home care typically receive services from multiple providers, making information retrieval and coordination an integral part of daily care. To ensure high-quality care for multimorbid clients in home care, providers across all interfaces must coordinate effectively, share information, and maintain continuity [15,25,26]. These activities can be conceptualised as health information management (HIM), encompassing tasks such as information retrieval, verification, and coordination across organisations [27,28]. This study addresses this gap by quantifying the time HCPs in Finnish home care spend on HIM tasks and examining factors associated with variation.

Our main research questions are:

1. How much time do HCPs spend weekly on HIM-related tasks?
2. Do HCPs’ occupational or demographic variables affect time use?

## Methods

### Study design

This exploratory cross-sectional online survey study was conducted between May and September 2024 to examine how home care professionals allocate their weekly working time to HIM-related tasks.

### Setting and participants

The survey targeted all home care professionals working in four publicly funded home care organizations in Finland. Eligible participants included practical nurses, registered nurses, physicians, nutritionists, physiotherapists, and other staff engaged in direct client care. In Finnish home care, services are typically organized as multiprofessional teams, with practical nurses and registered nurses forming the core team, supported by other professionals such as physicians and physiotherapists. A description of professional roles in Finnish home care is provided in Supplementary file 1.

In Finland, the social and healthcare services are organized by 21 regional wellbeing service counties. We selected four wellbeing service counties (referred to as regions A, B, C, and D later in the text) to ensure a balanced and nationally relevant sample. The regions varied in organization size, population density (9–117 persons/km²), geographic area (4,000–19,000 km²), and proportion of residents aged ≥75 years (9–14%). The proportion of ≥75-year-olds receiving home care services ranged from 11.3% to 15.1% [29]. Their home care units had between 450 and 1,700 employees and collectively covered about 22% of Finland’s 113,000 long-term home care clients [30]. Detailed differences are presented in Supplementary file 2.

The Finnish health and social care reform seeks to strengthen vertical integration. Before the 2023 reform, integration levels varied across regions due to historical and structural differences [31]. In regions A and C, municipalities had already established joint municipal authority with shared EHR systems and unified care coordination protocols, while municipalities in regions B and D operated independently (Table 1) [32]. As a result, regions B and D are still integrating municipal services into unified organizations (e.g., working on EHR interoperability). Together, these differences provide essential context for understanding variation in HIM time use and highlight how structural integration trajectories can influence the usability of HIM systems.

**Table 1:** Descriptions and characteristics of the four regions based on a national database [29] and information received from the regions. *Regional joint health and social care authority.


REGION	A	B	C	D

Short description	Smallest and most rural population base	Most urban, densely populated area	Rural region with ageing population	Mixed urban–rural region

Home care coverage among 75-year-olds (%)	15.1	11.3	12.7	13.4

Home care clients (% of total population)	2.7	1.3	2.2	2.0

Integration prior to the 2023 reform?*	Yes	No	Yes	No

Home care employees	< 500	> 1500	500–999	1000–1500

EHR interoperability	Interoperable; Health and social care on same system	Not interoperable; multiple systems used without integration	Partially interoperable; health and social care are on different modules	Not interoperable; multiple systems used without integration

Care coordination structures	Unified care coordination protocols across region	Some standardised care pathways but not used across region	Some standardised care pathways but not used across region	Some standardised care pathways but not used across region

Estimated integrated care maturity	High – Shared EHRs and multiple joint practices in place	Moderate/Low – Functional integration developing, but not uniform	Moderate – Structural separation hinders integration	Low – Vertical integration largely absent


### Survey instrument and variables

The time use items were part of a broader questionnaire developed to investigate HIM-related challenges in home care. The full instrument included 72 core items, with some items using branching logic, resulting in a total of up to 138 questions. Most items were measured on scales, and responses were mandatory to proceed. The questionnaire was developed based on findings from a prior qualitative study [12] and refined through six cognitive interviews with practical nurses, registered nurses, and physicians experienced in home care.

#### Time-use tasks

To assess time spent on HIM, respondents were presented with five predefined tasks. Each survey item used the same phrasing for each task: “How much time have you spent on each of these activities during the past 7 days?” The tasks covered three HIM domains—information retrieval, care coordination, and communication—and were as follows:

Q1) Searching for information in the client’s record or information system needed for their care.
Q2) Coordinating the client’s care and services between different providers.
Q3) Contacting the client or their family to ask about care-related information.
Q4) Responding to contact from the client or their family regarding care-related information.
Q5) Responding to contact from other individuals regarding care-related information.

These tasks may involve activities both within home care and across organizational boundaries, as multimorbid clients typically receive services from multiple providers. Responses for each question were predefined on a categorical scale: 0 minutes, 1–29 minutes, 30–59 minutes, 1–2 hours, 2–3 hours, 3–5 hours, and over 5 hours per week. The full version of the survey questions is presented in Supplementary file 3.

#### Demographic characteristics

Respondents provided background information on their demographic and professional characteristics. They were asked to report their profession (practical nurse, registered nurse, physician, therapist, assistant personnel, other personnel, explanations in Supplementary file 1), age, gender, years of experience in the health and social care sector, weekly working hours, and whether they participated in direct client care or held a supervisory position. Most of these questions included predefined answer categories, with an option for open-text responses when appropriate.

### Data collection and management

A self-selection sampling strategy was used. Designated local managers and supervisors distributed information about the study and a link to the survey through internal communication channels to HCPs. Participation was voluntary, and those interested could choose to respond. No specific eligibility criteria were applied. All professionals who chose to participate and submitted responses were included, regardless of whether they completed the full questionnaire.

In each region, the survey remained open for four weeks. To improve participation, we actively monitored response rates and sent reminder emails during the second and third weeks. While no financial incentives were provided, respondents could complete the survey during work hours. The data were collected and managed using REDCap electronic data capture tools (version 14.5.25; [33]), hosted at the University of Helsinki.

### Data analysis

We used descriptive statistics to examine the distribution of data, considering frequency, mean, and standard deviation. For the analyses, we reformatted the categorized time-use data into approximate means (in minutes) as follows: 0 (0 minutes), 15 (1–29 minutes), 45 (30–59 minutes), 90 (1–2 hours), 150 (2–3 hours), 240 (3–5 hours), and 300 (>5 hours). When time use is expressed as a proportion of working hours, the calculation is based on routine 7.5-hour shifts (total working hours 37.5h/week). For each respondent, we summed these means together to obtain the total time use. Responses marked as “Not included in my responsibilities” were recorded as not applicable. If all five questions were answered as not applicable, the participant was excluded from the analysis.

To better understand the variation within subgroups based on our control variables, we first analyzed variance (ANOVA) to explore differences between groups. We then built a multiple linear regression model using stepwise selection to identify factors associated with HIM time use [34]. This approach was chosen to support an exploratory examination of which factors were associated with HIM time use, without assuming predetermined relationships between variables. The model’s performance was assessed with adjusted *R*², non-adjusted *R*², and residual analysis. To reduce model complexity, we combined some of the smaller groups: “Other assisting staff” was grouped under “Other professions,” individuals <25 years were combined with those <30 years, individuals with <2 years and 2–4 years of experience were merged into a single <5 years group, and responses of “I do not know” were treated as “No.” A *P* value of <0.05 was used to determine statistical significance. Confidence intervals (95%) are reported for all regression coefficients.

Finally, we assessed the time use for each of the five tasks in detail by calculating the mean, standard deviation, and proportion for each task. For readability, we also converted the total times into hours and minutes. We used R (version 4.4.1) [35] for all analyses.

### Ethics and consent

Ethical approval was provided by the Ethics Committee of the Medical Faculty of the University of Helsinki (10/2023, dated 22.11.2023). At the start of the survey, participants were informed about the study’s voluntary nature, confidentiality of data, and their right to withdraw from the study at any time. Informed consent was obtained electronically before participants could proceed to the survey.

### Patient and public involvement

No patients or members of the general public were involved in the design or conduct of this study. However, home care professionals participated in the development of the survey instrument through cognitive interviews, which focused on improving question clarity and relevance. These interviews focused on clarity and phrasing and were not included in the final dataset.

## Results

### Characteristics of study participants

The analysis included 625 responses from a total of 638. Thirteen responses where the answer to all five questions was “Not included in my work” were excluded. Response rates ranged from 14.2% to 21.3% between regions, with a weighted average of 17.1%. Most respondents were practical nurses (67.4%) and registered nurses (19.8%), in full-time work (90.7%), and engaged in direct care of home care clients (90.2%) (Table 2). The respondents’ age distribution leaned toward the older age groups, 50–59-year-olds being the largest age group (27.0%). Most of the respondents were women (90.9%). The distribution of years of experience was relatively even. A minority of respondents were in a supervisory role (7.1%). The distribution of professionals, age groups, and years of experience varied across different regions. The main difference was in the proportions of different professional groups. Region A, which had the smallest sample, showed a higher proportion of practical nurses compared with other regions, resulting in smaller proportions of other professional groups.

**Table 2:** Description of respondents’ characteristics and variables.


VARIABLE	REGION A n (%)	REGION B n (%)	REGION C n (%)	REGION D n (%)	TOTAL n (%)

Respondents	96	142	146	241	625

Profession					

Practical nurse	76 (79.2)	85 (59.9)	104 (71.2)	156 (64.7)	421 (67.4)

Registered nurse	16 (16.7)	34 (23.9)	25 (17.1)	49 (20.3)	124 (19.8)

Physician	1 (1.0)	6 (4.2)	3 (2.1)	9 (3.7)	19 (3.0)

Therapist	1 (1.0)	7 (4.9)	1 (0.7)	3 (1.2)	12 (1.9)

Other	2 (2.1)	10 (7.0)	13 (8.9)	24 (10.0)	49 (7.8)

Age					

<30	16 (16.7)	19 (13.4)	22 (15.1)	38 (15.8)	95 (15.2)

30–39	19 (19.8)	34 (23.9)	30 (20.5)	64 (26.6)	147 (23.5)

40–49	24 (25.0)	37 (26.1)	37 (25.3)	56 (23.2)	154 (24.6)

50–59	30 (31.3)	46 (32.4)	37 (25.3)	56 (23.2)	169 (27.0)

≥60	7 (7.3)	6 (4.2)	20 (13.7)	27 (11.2)	60 (9.6)

Gender					

Women	85 (88.5)	128 (90.1)	139 (95.2)	216 (89.6)	568 (90.9)

Men	10 (10.4)	11 (7.7)	6 (4.1)	19 (7.9)	46 (7.4)

Other and NA	1 (1.0)	3 (2.1)	1 (0.7)	6 (2.5)	11 (1.8)

Years of experience					

<5	21 (21.9)	30 (21.1)	21 (14.4)	42 (17.4)	114 (18.2)

5–9	24 (25.0)	29 (20.4)	38 (26.0)	48 (19.9)	139 (22.2)

10–14	14 (14.6)	25 (17.6)	29 (19.9)	49 (20.3)	117 (18.7)

15–19	5 (5.2)	17 (12.0)	16 (11.0)	25 (10.4)	63 (10.1)

20–24	12 (12.5)	18 (12.7)	11 (7.5)	30 (12.4)	71 (11.4)

≥25	20 (20.8)	23 (16.2)	31 (21.2)	47 (19.5)	121 (19.4)

Role-related status					

Full-time employment (Yes)	88 (91.7)	127 (89.4)	135 (92.5)	217 (90.0)	567 (90.7)

Supervisory role (Yes)	6 (6.3)	10 (7.0)	18 (12.3)	10 (4.1)	44 (7.0)

Direct care (Yes)	81 (84.4)	124 (87.3)	131 (89.7)	228 (94.6)	564 (90.2)


### Total time use on health information management

On average, the respondent spent a weekly total of 4 hours and 18 minutes on HIM (258 minutes, SD = 278), with a median of 2 hours and 45 minutes (165 minutes; interquartile range 75–315 minutes). During a typical 7.5-hour shift, HCPs spent an average of 52 minutes (11.5%) and at the median 33 minutes (7.3%) on HIM tasks. The total time varied between 0 and 25 hours, corresponding to the minimum and maximum response options in our survey.

**Figure 1:**
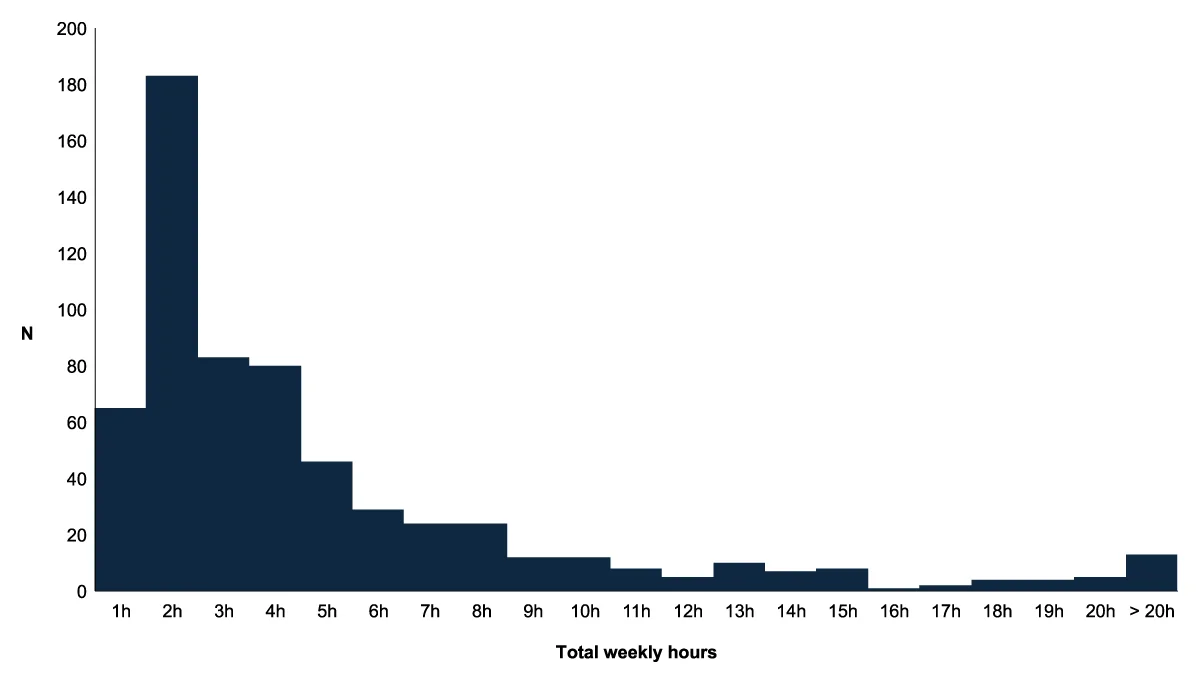
Distribution of total weekly time spent on health information management. N = 625. Responses exceeding 20 hours (n = 13) were aggregated for visualization because of the relatively low number of such responses.

The distribution of total time is presented in Figure 1.

### Distribution of time spent on health information management by profession and tasks

The average total time spent on HIM varied significantly both between and within professions for all five tasks (Table 3). Registered nurses spent the most time on almost every task, resulting in a total time of 8 hours and 38 minutes (518 minutes). Physicians spent 5 hours and 36 minutes (336 minutes) and therapists 5 hours and 10 minutes (310 minutes), while practical nurses and other HCPs spent approximately 3 hours.

**Table 3:** Weekly average time spent on health information management for each task by profession.


TASK	PRACTICAL NURSE (n = 421)		REGISTERED NURSE (n = 124)		PHYSICIAN (n = 19)		THERAPIST (n = 12)		OTHER (n = 49)	
				
	*MEAN (±SD)*	*% OF TOTAL*	*MEAN (±SD)*	*% OF TOTAL*	*MEAN (±SD)*	*% OF TOTAL*	*MEAN (±SD)*	*% OF TOTAL*	*MEAN (±SD)*	*% OF TOTAL*

Q1. Retrieving information	60 (66)	32.2	137 (99)	25.7	164 (111)	46.5	108 (84)	32	65 (79)	32.7

Q2. Care coordination	35 (45)	18.6	117 (89)	22	44 (41)	12.5	90 (78)	26.7	37 (47)	18.6

Q3. Contacting clients	30 (42)	16	93 (77)	17.5	58 (84)	16.3	40 (27)	11.9	34 (53)	17.1

Q4. Responding to client contact	31 (40)	16.6	92 (80)	17.3	40 (59)	11.3	54 (81)	16	34 (51)	17.1

Q5. Inter-professional communication	31 (46)	16.7	93 (86)	17.4	48 (86)	13.5	45 (46)	13.4	29 (43)	14.6

Average total minutes	185 (195)		518 (373)		336 (271)		310 (208)		182 (224)	

Average total; h, minutes	3 h 5 min		8 h 38 min		5 h 36 min		5 h 10 min		3 h 2 min	


N = 625. The average total was calculated by summing answers from Q1 to Q5; thus, it differs from the sum of the averages of each question. Missing values (answer option not applicable) for the questions are as follows: Q1 = 6, Q2 = 39, Q3 = 11, Q4 = 6, Q5 = 18. The full-length items are presented in Supplementary file 4.

Regarding time allocation for different tasks (Q1–Q5), registered nurses used the most time on each task, while practical nurses and other HCPs spent less time than therapists and physicians on all tasks. Retrieving information took most of the physicians’ time (164 minutes), corresponding to 47% of their total time on HIM. Registered nurses spent 117 minutes and therapists 90 minutes on care coordination, while other professions spent 35–44 minutes. Registered nurses spent on average 278 minutes contacting clients, responding to their inquiries, and engaging in interprofessional communication, while physicians spent 146 minutes, therapists 139 minutes, other professions 97 minutes, and practical nurses 92 minutes. Minimum values were 0 minutes across all questions and professions. Maximum values were 300 minutes, except for physicians (Q2 max = 150, Q4 max = 240) and other professions (Q2 max = 240, Q3 max = 240, Q5 max = 240). Information on response rates for each question categorized by region and profession is presented in Supplementary file 4.

### Variables affecting total time spent on health information management

We found that profession and direct patient care were the only statistically significant (P < 0.05) factors in our ANOVA analysis for the total HIM time (Table 4). We initially built our multiple linear regression model by including all 26 covariates (reduced list in Table 2), resulting in an R² of 0.222 (non-adjusted R² 0.248). We applied a stepwise reduction strategy, sequentially removing the least significant variables to improve model parsimony while preserving explanatory power. The final model retained 13 covariates, resulting in an adjusted R² of 0.229 (non-adjusted R² 0.241). Age, gender, years of experience, and supervisory role did not contribute significantly to our model, even after age and years of experience were grouped into broader categories.

**Table 4:** Descriptive statistics (mean and standard deviation), univariate analysis of variance (ANOVA), and multiple linear regression model results including 95% confidence intervals (CI) of weekly health information management time use.


VARIABLE	MEAN (SD)	UNIVARIATE p-VALUE, ANOVA	COEFFICIENT β, REG. MODEL	95% CI, REG. MODEL	pr(>|t|), REG. MODEL*

Intercept (reference)			510.73	446; 576	<0.001*

Profession		<0.001*			

Registered nurse (reference)	518 (373)				

Practical nurse	185 (195)		–333.65	–383; –284	<0.001*

Physician	336 (271)		–184.95	–303; –67	0.002*

Therapist	310 (208)		–210.10	–356; –64	0.005*

Other personnel	182 (224)		–331.65	–413; –250	<0.001*

Region		0.282			

A (reference)	222 (279)				

B	285 (293)		27.59	–37; 92	0.399

C	221 (239)		–7.52	–71; 56	0.16

D	278 (289)		33.30	–26; 92	0.267

Direct client care		0.041*			

Direct client care (Yes) (reference)	262 (287)				

Direct client care (No)	218 (176)		–69.51	–135; –4	0.038*

Full-time employment		0.316			

Full-time employment (Yes) (reference)	262 (279)				

Full-time employment (No)	220 (266)		–34.05	–101; 33	0.316


*Pr(>|t|) denotes the two-tailed p-value for a t-test under the null hypothesis that the coefficient is zero. Values in the table represent the mean total weekly HIM time (in minutes) for each subgroup independently, regardless of other variables. Adjusted R² = 0.229, non-adjusted R² = 0.241. Degrees of freedom = 615. P value = 2.2 × 10^–16^. Degrees of freedom = 615. P value = 2.2 × 10^–16^.

## Discussion

Among Finnish home care professionals, the time spent on HIM tasks was substantial, particularly for registered nurses, physicians, and therapists. Registered nurses reported the highest workload, averaging 1.5–2 hours daily on HIM, while physicians and therapists spent over 1 hour, representing 15–25% of their weekly working time (37.5 hours). These findings extend previous time use research [14,20,21,22,23], showing that home care professionals face a burden comparable to those in hospital-based EHR studies. Notably, HIM tasks alone constitute a substantial portion (15–25%) of the broader indirect work, as documented in a previous study related to Finnish home care time use [24]. From an integration perspective, this indicates that professionals operating at care interfaces shoulder a disproportionate load, reflecting persistent gaps in vertical integration. To our knowledge, this is the first study to provide task-level and profession-comparative evidence on HIM time use in home care.

Information retrieval and coordination tasks accounted for the largest share of HIM time, especially for registered nurses, physicians and therapists. In contrast, practical nurses, whose roles involve fewer cross-organisational interactions, spent much less time on these tasks. Regression analysis further showed that professionals not directly working with clients spent approximately 70 fewer minutes on HIM tasks, supporting the finding that direct client contact is associated with higher HIM burden. This pattern supports earlier findings that professionals working at organizational interfaces face the greatest administrative demands [36]. Our task-level analysis adds new insight by specifying which activities drive this workload and linking them to professions. In outpatient care, physicians have been shown to spend nearly half of their working time on EHR-related tasks [37,38], which aligns with our findings even though our focus was on retrieval and coordination rather than documentation. We also refine earlier Finnish home care studies [14] by showing that within indirect patient work, information retrieval and coordination are the most time-intensive elements.

High HIM time use can be interpreted in two contrasting ways. On one hand, it may represent value-adding time invested in purposeful coordination, structured communication, and shared decision-making that support continuity of care [39]. On the other hand, it may signal inefficiencies, such as fragmented information across multiple systems, unclear responsibilities, or poor interoperability that increase workload and reduce efficiency, which is widely acknowledged in the Finnish EHR systems [40,41]. Distinguishing between these two forms of time use is critical for assessing the potential to improve efficiency in information gathering and care coordination.

There were no statistically significant differences between the four Finnish regions, despite their differing levels of integration maturity [31,32]. This contrasts with expectations from integration maturity assessments, which suggest clear differences between regions at different levels of integration maturity [31]. Based on recent national studies documenting substantial variation in EHR system quality and user satisfaction across the four regions [41,42], we expected such differences to emerge. One explanation is that workload pressures impose implicit time limits on HIM tasks [43], forcing professionals to complete similar tasks regardless of system quality, contributing to documented patient safety concerns when systems fail to support workflow adequately [41]. Additionally, some parts of core documentation and information retrieval requirements are nationally standardized, mandating similar tasks across all regions. Stronger integration may therefore not reduce time spent, but rather improve information quality and continuity, whereas weaker integration compromises it.

Given that EHR systems are central tools for HIM in modern health and social care [44], it is notable that most prior studies have focused on overall EHR use [20] rather than specific tasks or profession-comparison. Our findings extend this research by showing that different professions face distinct HIM demands, underscoring the need for role-sensitive system design. Addressing these demands could reduce unnecessary workload and allow more time for direct client care. We found no other demographic or occupational factors associated with time use, and our regression model explained only 23% of the variance. This indicates that unmeasured factors, such as EHR usability, local workflows, and team communication norms, may play a substantial role. Self-reported measures may also have led to over- or underestimation, depending on whether tasks were perceived as disruptive or routine.

### Strengths and weaknesses

This exploratory study offers insights into the time spent by different professions in the outpatient care of multimorbid clients, aligning with and extending previous research [37,38,45]. As the first study to quantify HIM time across professional roles in home care, it provides valuable descriptive data on an understudied area. Similar administrative burdens and information continuity challenges have been reported internationally, particularly in North America [7,37,38,45,46] and Europe [4,6,16,23,26,47,48,49,50]. Our data from four Finnish regions capture variation in integration maturity, EHR systems, and coordination structures, enhancing generalizability.

Important limitations must be acknowledged. Response rates (17%) were adequate for large organizational surveys but raise concerns about selection bias, as those most burdened by HIM tasks or most time-constrained may be differentially represented among respondents. Time use was self-reported retrospectively, a method with known systematic biases: comparative studies have documented that self-report tends to over-estimate direct patient activities while under-estimating fragmented administrative tasks, which may affect both the magnitude and distribution of our findings [51]. Additionally, our measurement does not distinguish between HIM tasks driven by integration gaps versus routine daily documentation. Given these methodological constraints, our findings should be interpreted as indicative of HIM workload patterns rather than precise time measurements.

### Practical implications for HIM system development

Our results highlight that HIM tasks, especially information retrieval, remain time-intensive regardless of integration maturity. This indicates that reforms or maturity gains alone are insufficient, and integration efforts should prioritise operational systems and day-to-day workflows. The heaviest demands were observed among professionals working at organisational interfaces, where vertical integration is weakest, and gaps in responsibilities and information continuity are most visible. Integration strategies should therefore focus on role-sensitive system design and workflow support at these interfaces.

Reflecting on our findings, we hypothesize that integration should focus more on professional roles and tasks, and on how both normative and functional integration can be developed to support each role. EHRs and other health information systems could play a central role in enabling continuity, especially in home care where staff turnover is high and multiple providers are involved. However, current systems seem to prioritize fulfilling legislative requirements and managerial functions such as documenting daily work and clinical tasks rather than offering applications or tools for the coordination of care across providers [52]. Strengthening functional integration at care interfaces could partially offset the challenges of sustaining normative integration in high-turnover environments.

Health information systems have moved from paper documentation to larger pools of electronic client data [19], which requires efficient compilation and provision of care-related data. Yet many systems suffer from design flaws [49,53], poor interoperability [50], and usability challenges [48]. These shortcomings can create information overload, complicate decision-making [46,47], and may potentially compromise patient safety [17]. Such weaknesses may translate into invisible time use on HIM and partly explain the high time use in this study, especially among professionals working at organizational interfaces.

At the same time, EHR systems can provide clear benefits when well designed: optimized systems improve care [54], and better-structured data entry could further support this [55]. As client data grows exponentially, EHRs should evolve from digital repositories into platforms that integrate information across providers and time frames. Such models have been proposed [52], but implementation remains limited. Achieving this will require coordination tools that are distinct from those used in direct patient care, ensuring that information flows seamlessly across organisational boundaries. Natural language processing could reduce documentation complexity, summarise care histories, and tailor information to professional roles [56]. AI-assisted tools could further support this transition, though current development in home care has focused primarily on documentation and information generation, such as automated summarisation and structured data entry, rather than broader information integration. Peer-reviewed evidence on their benefits remains limited.

### Future research

Future research should move beyond measuring overall HIM time to identify which tasks create value and which represent waste. An important area for examination is what share of routine documentation offers clinical value. While it’s obvious there is a certain minimum to ensure patient safety and sharing information needed for daily care of the clients, some mandatory HIM tasks may consume substantial time in relation to the achieved benefit. Given that only 15% of physicians agreed that their EHR system provides a summary view facilitating a comprehensive picture of the patient’s situation [41], there is a clear need for systems that better synthesize and present information to support clinical decision-making. While our study shows which professions devote the most time, further work is needed to analyse the underlying causes and whether inefficient tasks can be reduced. Such studies would clarify where integration gaps generate unnecessary effort and how EHR systems and workflows should be redesigned to make essential data more accessible. Combining self-reports with observational methods or EHR log data would provide more robust evidence. Research should also extend beyond hospitals to home and community care, with particular attention to multimorbid patients whose complex needs will increasingly define the future of integrated care.

## Conclusions

In conclusion, HIM is time-consuming and accounts for 15–25% of HCPs’ working time, especially among registered nurses, physicians, and physiotherapists, whose roles involve more frequent interaction with other health care providers in addition to home care. Information retrieval was the most time-consuming task for each profession, but a significant amount of time was also spent on communication about care and care coordination. Higher HIM time use was observed among professionals whose roles involve more cross-organizational interaction, which may reflect gaps in vertical integration. Our findings further indicate that variation in time use is shaped more by professional roles than by regional integration maturity.

These findings suggest the potential value of strengthening both functional integration (e.g., interoperable and user-friendly EHRs) and normative integration (e.g., shared goals and collaboration practices) to ensure that essential information flows seamlessly across providers and care levels. Strengthening integration in this way may reduce inefficiencies and allow more time to be directed towards direct client care. This study provides novel task-level evidence from home care, a setting where HIM time use has been largely understudied. Further research should focus on producing more specific results and on advancing health information systems that better support the care of multimorbid individuals.

### Key learnings

- Home care professionals spend 3 to 8.5 hours weekly on health information management
- Profession was the only factor associated with time use, with registered nurses, physicians, and therapists spending more time than other professionals
- Information retrieval accounted for at least one third of the total time used
- Current information systems require further development to better support integrated, complex care for multimorbid clients

## Additional Files

The following additional files are available for this article:

10.5334/ijic.9881.s1 Supplementary File 1. Table A1. Descriptions of professions in Finnish home care.

10.5334/ijic.9881.s2 Supplementary File 2. Table B1. Region-related information from public sources.

10.5334/ijic.9881.s3 Supplementary File 3. Table C1. Questionnaire survey form, adapted from the original online version in Finnish.

10.5334/ijic.9881.s4 Supplementary File 4. Table D1. Response rates for each question and the distribution of answers by region and profession.

## Data Availability

The datasets used and/or analysed during the current study are available from the corresponding author on reasonable request.
