# Supplementary material for: Time Well Spent? Time Use for Health Information Management Across Home Care Roles: A Cross-Sectional Survey Study in Finland
Source: Int J Integr Care. 2026 Sep 8;26(3):17. doi: 10.5334/ijic.9881 (PMC13564982; doi:10.5334/ijic.9881)
Supplement: Supplementary file 1 [file ijic-26-3-9881-s1.pdf]

SUPPLEMANTARY APPENDIX

Supplement to: Time Well Spent? Time Use for Health Information Management Across Home Care Roles: A Cross-Sectional Survey Study in Finland

This appendix has been provided by the authors to give readers additional information about the work.

SUPPLEMENTARY FILE 1

A1. Descriptions of professions in Finnish home care.

**Profession category**	**Professions (Finnish term)**	**Primary role in home care**
Practical nurse	Practical nurse (lähihoitaja)	Conducts the majority of direct home visits; responsible for personal care and some nursing procedures.<br>Holds an upper secondary vocational qualification (approximately 3 years).
Registered nurse	Registered nurse (sairaanhoitaja)	Responsible for clinical oversight, specialised in clinical procedures (e.g. wound care), and care planning, may hold supervisory or team leadership responsibilities.<br>Holds a bachelor's degree from a university of applied sciences (3.5 years).
Physician	Physician (lääkäri)	Responsible for medical assessment, diagnosis, and treatment planning.<br>Holds a medical degree from a university (6 years) and possibly a specialist degree (5 to 6 years).
Therapist	Physiotherapist (fysioterapeutti); occupational therapist (toimintaterapeutti); nutritionist (ravitsemusterapeutti)	Responsible for assistive devices, rehabilitation, nutritional guidance, and functional capacity assessment.<br>Holds a bachelor’s degree from university of applied sciences (3.5 years), except for the nutritionist, who typically holds a master's degree from a university (5 years).
Other	Care assistants (hoiva-avustaja); other personnel (muu henkilöstö)	Supports daily care tasks under supervision; does not require profession-specific clinical training.<br>Educational background varies.

Social welfare professionals (e.g., social workers, gerontologists) are not listed as a separate category, as they were able to respond under “Other” if present in the participating organizations.
