# Supplementary material for: Time Well Spent? Time Use for Health Information Management Across Home Care Roles: A Cross-Sectional Survey Study in Finland
Source: Int J Integr Care. 2026 Sep 8;26(3):17. doi: 10.5334/ijic.9881 (PMC13564982; doi:10.5334/ijic.9881)
Supplement: Supplementary file 2 [file ijic-26-3-9881-s2.pdf]

SUPPLEMENTARY FILE 2

Table B1. Region-related information from public sources.

	Region A	Region B	Region C	Region D	National
Number of municipalities	9	10	13	27	309
Size of the region (km²)	5,000	4,000	19,000	11,000	304,000
Population	125,000	486,000	163,000	486,000	5,564,000
Population density (people/km²)	23.5	116.5	8.6	46	18.4
Proportion of the region classified as urban (%)	84.4	93.1	73.4	86.2	86.9
Number of university hospitals	0	1	0	1	5
Municipality-wide integration before reform	Yes	No	Yes	No	–
Estimated integration level of electronic health records systems (1–4)*	1	4	2	4	–
Population aged 75 years and older (% of total)	35,881 (14)	88,592 (9)	45,826 (13)	115,837 (12)	1,282,654 (11)
Total number of home care clients	4,855	11,382	7,405	18,353	194,963
… who are long-term clients (% of total)	3,257 (67)	7,156 (63)	3,789 (51)	10,672 (58)	115,052 (59)
… as a proportion of the total population (%)	3.9	2.3	4.5	3.8	3.5
Total number of long-term home care clients	3,257	7,156	3,789	10,672	115,052
… aged 75–84 years (% of total)	1,117 (34)	2,576 (36)	1,297 (34)	3,526 (33)	38,821 (34)
… aged 85 years and older (% of total)	1,497 (46)	2,906 (41)	1,576 (42)	4,731 (44)	49,444 (43)
Long-term home care visits	1,396,133	2,293,789	1,581,258	3,455,841	39,381,260
... made in person (house calls) (% of total)	1,240,696 (89)	2,161,870 (94)	1,417,475 (90)	3,310,293 (96)	37,121,466 (94)
… to clients aged 75–84 years (% of total)	460,913 (33)	760,635 (33)	524,501 (33)	1,106,393 (32)	12,722,491 (32)
… to clients aged 85 years and older (% of total)	678,320 (49)	1,103,335 (48)	729,348 (46)	1,741,613 (50)	19,243,807 (49)
Average number of long-term home care visits per client	429	321	417	324	342
… for clients aged 75–84 years	413	295	404	314	328
… for clients aged 85 years and older	453	380	463	368	389

* 1 = cohesive, 2 = mostly cohesive, 3 = partially fragmented, 4 = fragmented.
