# Supplementary material for: Time Well Spent? Time Use for Health Information Management Across Home Care Roles: A Cross-Sectional Survey Study in Finland
Source: Int J Integr Care. 2026 Sep 8;26(3):17. doi: 10.5334/ijic.9881 (PMC13564982; doi:10.5334/ijic.9881)
Supplement: Supplementary file 3 [file ijic-26-3-9881-s3.pdf]

SUPPLEMENTARY FILE 3

Table C1. Questionnaire survey form, adapted from the original online version in Finnish.

How much time have you spent on each of these activities during the past 7 days?

	0min	1–29min	30–59min	1–2h	2–3h	3–5h	Over 5h	NA
Q1. Retrieving information needed for the client's care in client and patient information systems								
Q2. Coordinating the client's care and services between different providers (e.g., scheduling appointments, contacting others, gathering information from other units)								
Q3. Contacting the client or their family to ask about care-related information								
Q4. Responding to contact from the client or their family regarding care-related information								
Q5. Responding to contact from other professionals regarding care-related information								

*NA = Not applicable (Not included in my responsibilities)
