# Supplementary material for: Time Well Spent? Time Use for Health Information Management Across Home Care Roles: A Cross-Sectional Survey Study in Finland
Source: Int J Integr Care. 2026 Sep 8;26(3):17. doi: 10.5334/ijic.9881 (PMC13564982; doi:10.5334/ijic.9881)
Supplement: Supplementary file 4 [file ijic-26-3-9881-s4.pdf]

SUPPLEMENTARY FILE 4

Table D1. Response rates for each question and the distribution of answers by region and profession.

How much time have you spent on each of these activities during the past 7 days?

Q1) Retrieving information needed for the client's care in client and patient information systems.

Region	Category	Other	Practical nurse	Therapist	Physician	Registered nurse	Total
A	0	1	1	0	0	0	2
	1–29min	0	31	0	0	3	34
	30–59min	0	24	0	0	1	25
	1–2h	0	9	0	0	3	12
	2–3h	0	7	1	0	4	12
	3–5h	1	3	0	1	4	9
	>5h	0	1	0	0	1	2
	NA*	0	0	0	0	0	0
B	0	0	7	0	0	2	9
	1–29min	5	36	1	1	6	49
	30–59min	1	12	2	1	3	19
	1–2h	1	12	4	1	6	24
	2–3h	0	10	0	0	8	18
	3–5h	0	8	0	2	1	11
	>5h	1	0	0	1	7	9
	NA*	2	0	0	0	1	3
C	0	0	2	0	1	0	3
	1–29min	4	53	0	2	5	64
	30–59min	1	15	1	0	3	20
	1–2h	5	22	0	0	1	28
	2–3h	2	7	0	0	9	18
	3–5h	0	5	0	0	3	8
	>5h	1	0	0	0	3	4
	NA*	0	0	0	0	1	1
D	0	2	7	0	0	0	9
	1–29min	12	69	0	0	7	88
	30–59min	3	26	0	0	7	36
	1–2h	2	30	1	1	13	47
	2–3h	2	13	0	3	3	21
	3–5h	1	8	1	2	14	26
	>5h	2	3	1	3	5	14
	NA*	0	0	0	0	0	0
Total		49	421	12	19	124	625

Q2) Coordinating the client's care and services between different providers (e.g., scheduling appointments, contacting others, gathering information from other units).

Region	Category	Other	Practical nurse	Therapist	Physician	Registered nurse	Total
A	0	0	12	0	0	0	12
	1–29min	1	44	1	0	3	49
	30–59min	0	8	0	1	1	10
	1–2h	0	7	0	0	6	13
	2–3h	0	1	0	0	2	3
	3–5h	1	0	0	0	3	4
	>5h	0	1	0	0	0	1
	NA*	0	3	0	0	1	4
B	0	1	14	0	0	1	16
	1–29min	3	28	1	1	6	39
	30–59min	2	21		1	5	29
	1–2h	1	9	2	3	6	21
	2–3h	1	2	1	0	2	6
	3–5h	0	3	0	0	9	12
	>5h	0	0	0	0	3	3
	NA*	2	8	3	1	2	16
C	0	2	13	0	0	0	15
	1–29min	4	58	0	3	5	70
	30–59min	3	22	1	0	5	31
	1–2h	3	5	0	0	5	13
	2–3h	0	2	0	0	5	7
	3–5h	0	1	0	0	3	4
	>5h	0	0	0	0	1	1
	NA*	1	3	0	0	1	5
D	0	3	22	0	1	1	27
	1–29min	11	62	1	3	6	83
	30–59min	3	43	0	2	8	56
	1–2h	1	16	0	0	14	31
	2–3h	0	5	1	1	10	17
	3–5h	0	2	1	0	6	9
	>5h	0	1	0	0	3	4
	NA*	6	5	0	2	1	14
Total		49	421	12	19	124	625

Q3) Contacting the client or their family to ask about care-related information.

Region	Category	Other	Practical nurse	Therapist	Physician	Registered nurse	Total
A	0	1	15	0	1	1	18
	1–29min	0	48	1	0	2	51
	30–59min	0	10	0	0	3	13
	1–2h	0	2	0	0	5	7
	2–3h	0	0	0	0	2	2
	3–5h	1	0	0	0	2	3
	>5h	0	1	0	0	0	1
	NA*	0	0	0	0	1	1
B	0	2	12	0	0	1	15
	1–29min	2	44	3	3	9	61
	30–59min	4	17	3	1	4	29
	1–2h	1	5	1	1	8	16
	2–3h	0	5	0	0	6	11
	3–5h	0	1	0	0	3	4
	>5h	0	0	0	1	1	2
	NA*	1	1	0	0	2	4
C	0	1	19	0	1	0	21
	1–29min	8	59	0	2	5	74
	30–59min	3	17	1	0	5	26
	1–2h	0	6	0	0	9	15
	2–3h	0	3	0	0	3	6
	3–5h	1	0	0	0	0	1
	>5h	0	0	0	0	2	2
	NA*	0	0	0	0	1	1
D	0	5	17	0	2	0	24
	1–29min	13	84	1	1	8	107
	30–59min	2	37	1	2	13	55
	1–2h	1	9	1	2	16	29
	2–3h	1	4	0	0	3	8
	3–5h	0	2	0	1	7	10
	>5h	0	2	0	0	1	3
	NA*	2	1	0	1	1	5
Total		49	421	12	19	124	625

Q4) Responding to contact from the client or their family regarding care-related information.

Region	Category	Other	Practical nurse	Therapist	Physician	Registered nurse	Total
A	0	0	16	1	0	1	18
	1–29min	1	47	0	1	2	51
	30–59min	0	11	0	0	3	14
	1–2h	0	1	0	0	5	6
	2–3h	1	0	0	0	2	3
	3–5h	0	0	0	0	1	1
	>5h	0	1	0	0	1	2
	NA*	0	0	0	0	1	1
B	0	2	11	0	1	1	15
	1–29min	2	43	5	3	9	62
	30–59min	3	21	1	1	6	32
	1–2h	3	7	1	1	7	19
	2–3h	0	2	0	0	7	9
	3–5h	0	1	0	0	3	4
	>5h	0	0	0	0	1	1
	NA*	0	0	0	0	0	0
C	0	3	13	0	1	0	17
	1–29min	5	60	0	2	9	76
	30–59min	3	19	1	0	6	29
	1–2h	1	7	0	0	5	13
	2–3h	0	4	0	0	2	6
	3–5h	0	0	0	0	1	1
	>5h	1	0	0	0	1	2
	NA*	0	1	0	0	1	2
D	0	4	16	0	2	1	23
	1–29min	16	78	0	2	7	103
	30–59min	2	40	2	1	11	56
	1–2h	1	13	0	2	17	33
	2–3h	0	5	0	0	5	10
	3–5h	0	2	0	1	5	8
	>5h	0	1	1	0	3	5
	NA*	1	1	0	1	0	3
Total		49	421	12	19	124	625

Q5) Responding to contacts from other professionals regarding care-related information.

Region	Category	Other	Practical nurse	Therapist	Physician	Registered nurse	Total
A	0	0	18	0	0	0	18
	1–29min	1	45	1	1	2	50
	30–59min	0	12	0	0	3	15
	1–2h	1	0	0	0	6	7
	2–3h	0	0	0	0	0	0
	3–5h	0	0	0	0	2	2
	>5h	0	1	0	0	2	3
	NA*	0	0	0	0	1	1
B	0	1	17	1	2	1	22
	1–29min	5	38	3	2	9	57
	30–59min	2	12	0	2	6	22
	1–2h	1	9	2	0	8	20
	2–3h	0	2	0	0	5	7
	3–5h	0	2	0	0	1	3
	>5h	0	1	0	0	3	4
	NA*	1	4	1	0	1	7
C	0	1	22	0	0	1	24
	1–29min	8	52	0	3	10	73
	30–59min	2	15	1	0	5	23
	1–2h	0	9	0	0	3	12
	2–3h	1	2	0	0	3	6
	3–5h	1	0	0	0	2	3
	>5h	0	1	0	0	1	2
	NA*	0	3	0	0	0	3
D	0	6	25	0	1	2	34
	1–29min	13	71	1	3	6	94
	30–59min	2	34	1	1	15	53
	1–2h	1	16	0	0	14	31
	2–3h	0	4	1	0	5	10
	3–5h	0	1	0	1	4	6
	>5h	0	2	0	1	3	6
	NA*	2	3	0	2	0	7
Total		49	421	12	19	124	625

*NA = Not applicable (Not included in my responsibilities)
